# Supplementary material for: Risk of Post-COVID-19 Uveitis and Risk Modification by Vaccination: A Nationwide Retrospective Cohort Study
Source: Vaccines (Basel). 2024 Jun 6;12(6):631. doi: 10.3390/vaccines12060631 (PMC11209449; doi:10.3390/vaccines12060631)
Supplement: Supplementary file 1 [file vaccines-12-00631-s001.zip › vaccines-3027884-supplementary.pdf]

Table S1. Definitions and corresponding ICD-10 and Korean Classification of Disease (KCD)-7 or -8 codes for uveitis types and observation periods

Category	ICD-10 codes	KCD codes	Specific conditions
**Uveitis**			
Noninfectious uveitis	H20.0	H20.0	Acute and subacute iridocyclitis
	H20.1	H20.1	Chronic iridocyclitis
	H20.2	H20.2	Lens-induced iridocyclitis
	H20.8	H20.8	Other iridocyclitis
	H20.9	H20.9	Iridocyclitis, unspecified
	H22.1	H22.1	Iridocyclitis in other diseases classified elsewhere
	H22.8	H22.8	Other disorders of iris and ciliary body in diseases classified elsewhere
	H30.0	H30.0	Focal chorioretinal inflammation
	H30.1	H30.1	Disseminated chorioretinal inflammation
	H30.2	H30.2	Posterior cyclitis
	H30.8	H30.8	Other chorioretinal inflammations
	H30.9	H30.9	Chorioretinal inflammation, unspecified
	H35.06	H35.05	Retinal vasculitis
Infectious uveitis	H22.0	H22.0	Iridocyclitis in infectious and parasitic diseases classified elsewhere
	H32.0	H32.0	Chorioretinal inflammation in infectious and parasitic diseases classified elsewhere
Anterior uveitis	H20.0	H20.0	Acute and subacute iridocyclitis
	H20.1	H20.1	Chronic iridocyclitis
	H20.2	H20.2	Lens-induced iridocyclitis
	H20.8	H20.8	Other iridocyclitis
	H20.9	H20.9	Iridocyclitis, unspecified
	H22.1	H22.1	Iridocyclitis in other diseases classified elsewhere
	H22.8	H22.8	Other disorders of iris and ciliary body in diseases classified elsewhere

	H22.0	H22.0	Iridocyclitis in infectious and parasitic diseases classified elsewhere
Non-anterior uveitis	H30.0	H30.0	Focal chorioretinal inflammation
	H30.1	H30.1	Disseminated chorioretinal inflammation
	H30.2	H30.2	Posterior cyclitis
	H30.8	H30.8	Other chorioretinal inflammations
	H30.9	H30.9	Chorioretinal inflammation, unspecified
	H35.06	H35.05	Retinal vasculitis
	H32.0	H32.0	Chorioretinal inflammation in infectious and parasitic diseases classified elsewhere

**Observation periods**	**Definitions**
Pre-infection or vaccination	The period preceding any COVID-19 vaccination or infection event, whichever came first
Post-vaccination-only	The time following the first vaccination but preceding the first COVID-19 infection event
Post-infection-only	The time following the first COVID-19 infection but preceding the first COVID-19 vaccination
Post-vaccination & infection	The period following both vaccination and infection, irrespective of the sequence of occurrence

Table S2. Risk of post-COVID-19 uveitis in four age groups in various periods before and after COVID-19 vaccination and infection

Period	Events				Rates				HR (95% CI)			
	<40	40-59	60-79	≥80	<40	40-59	60-79	≥80	<40	40-59	60-79	≥80
Pre-infection/vaccination	31,121	50,875	70,316	20,119	0.024	0.023	0.022	0.019	1.00 (ref)			
Post-vaccination only	496	914	1,238	234	0.024	0.023	0.023	0.022	1.11 (1.02-1.22)	1.15 (1.08-1.23)	1.15 (1.09-1.22)	1.43 (1.25-1.62)
Post-infection only	91	30	27	1	0.027	0.029	0.034	0.016	1.32 (1.07-1.62)	1.61 (1.13-2.30)	1.92 (1.32-2.80)	N/A*
Post-vaccination & infection	412	591	609	88	0.022	0.021	0.021	0.02	0.98 (0.89-1.08)	1.09 (1.00-1.18)	1.09 (1.00-1.18)	1.27 (1.03-1.56)

*Not applicable (unreliable) due to a small number (1) of event.

Table S3. Risk of post-COVID-19 uveitis in male and female patients in various periods before and after COVID-19 vaccination and infection

**Period**	**Events**		**Rates**		**HR (95% CI)**	
	Male	Female	Male	Female	Male	Female
Pre-infection or vaccination	79,609	92,822	0.023	0.022	1.00 (ref)	
Post-vaccination only	1,300	1,582	0.022	0.023	1.13 (1.07-1.19)	1.22 (1.16-1.28)
Post-infection only	68	81	0.027	0.029	1.54 (1.21-1.95)	1.66 (1.34-2.07)
Post-vaccination & infection	812	888	0.021	0.022	1.07 (1.00-1.15)	1.13 (1.06-1.20)
